# Comparison of Acute and Chronic Stage Ischemic Stroke Metabolome with Controls

**DOI:** 10.21203/rs.3.rs-2515376/v1

**Published:** 2023-01-30

**Authors:** Evgeny V. Sidorov, Madhusmita Rout, Chao Xu, Jordan Larsen, Evan Fields, Blair Apple, Kyle Smith, David Gordon, Juliane Chainakul, Dharambir Sanghera

**Affiliations:** University of Oklahoma Health Sciences Center; University of Oklahoma Health Sciences Center; University of Oklahoma Health Sciences Center; Duke University; University of Oklahoma Health Sciences Center; University of Oklahoma Health Sciences Center; University of Oklahoma Health Sciences Center; University of Oklahoma Health Sciences Center; University of Oklahoma Health Sciences Center; University of Oklahoma Health Sciences Center

**Keywords:** Metabolomics, ketone bodies, ischemic stroke, oxidative stress, pyruvate

## Abstract

**Background:**

Acute Ischemic Stroke (AIS), a major cause of disability, was previously associated with multiple metabolomic changes, but many findings were contradictory. Case-control and longitudinal study designs could have played a role in that. To clarify metabolomic changes, we performed a simultaneous comparison of ischemic stroke metabolome in acute, chronic stages of stroke and controls.

**Methods:**

Through the nuclear magnetic resonance (NMR) platform, we evaluated 271 serum metabolites from a cohort of 297 AIS patients in acute and chronic stages and 159 controls. We used Sparse Partial Least Squares-Discriminant analysis (sPLS-DA) to evaluate group disparity; multivariate regression to compare metabolome in acute, chronic stages of stroke and controls; and mixed regression to compare metabolome acute and chronic stages of stroke. We applied false discovery rate (FDR) to our calculations.

**Results:**

The sPLS-DA revealed separation of the metabolome in acute, chronic stages of stroke and controls. Regression analysis identified 38 altered metabolites. Ketone bodies, branched-chain amino acids (BCAAs), energy, and inflammatory compounds were elevated in the acute stage, but declined in the chronic stage, often to the same levels as in controls. Levels of other amino acids, phosphatidylcholines, phosphoglycerides, and sphingomyelins mainly did not change between acute and chronic stages, but was different comparing to controls.

**Conclusion:**

Our pilot study identified metabolites associated with acute stage of ischemic stroke and those that are altered in stroke patients comparing to controls regardless of stroke acuity. Future investigation in a larger independent cohort is needed to validate these findings.

## Introduction

Acute ischemic stroke (AIS) is a leading cause of serious disability in the United States^1^. Many attempts to improve our understanding of stroke pathophysiology and identify metabolic cascades related to stroke did yield desired results. One of the novel methods investigating disease pathophysiology was untargeted metabolomic profiling, which carries ability to identify large number of low molecular weight metabolites in bodily fluids. Identified metabolites may serve as disease biomarkers and uncover new disease mechanisms. Many authors attempted to identify AIS biomarkers using metabolomics, but these results were commonly inconclusive and, sometimes, contradictory^2^. Several case-control studies showed few metabolomic distinctions between AIS patients and healthy controls such as amino acids, sphingolipids, however, lack of follow-up data made it impossible to conclude whether discovered metabolites were driven by stroke acuity or indicated baseline differences in the metabolism of stroke and non-stroke individuals^3,4^. Based on these findings, our group presented two longitudinal pilot studies comparing metabolome in the acute and chronic stages of stroke^5,6^. Their results demonstrated how certain metabolites change between acute and chronic stages, but could not conclude whether they were different from control group. Therefore, in the present study, we simultaneously evaluated the metabolome in the acute and chronic stages of ischemic stroke and in controls without stroke. Such an approach may help to differentiate metabolites driven by acute cerebral ischemia from those that have baseline changes in stroke individuals, and create foundation for future metabolome stroke research.

## Methods

### Study Participants

The study participants were part of our ongoing, institutional review board-approved, Metabolome in Ischemic Stroke Study (MISS), which investigates biomarkers using genomics, metabolomics, and other - omics technologies^2,6–8^. All stroke patients underwent magnetic resonance imaging (MRI) with diffusion-weighted imaging (DWI) sequence during admission for AIS definition. All patients were evaluated on admission with the National Institute of Health Stroke Scale (NIHSS; 0–42 points reflecting the least to the most severe neurological deficit) to define severity of acute neurological impairment from stroke^9^. During follow-up (chronic stage), we performed neurological examination and reviewed medical records to rule out recurrent stroke. After obtaining informed consent, we collected serum in the acute (within the first 72 hours) and chronic (3–6 months) stages of ischemic stroke, as by that time many inflammatory markers reach normal levels and could represent baseline^10,11^. Controls without stroke were recruited from the cardiology clinic and local blood institute. We excluded patients with (1) hemorrhagic conversion of ischemic stroke and formation of parenchymal hematoma defined by the Heidelberg bleeding classification^12^; (2) systemic infection at presentation or during admission with fever > 38.0 C, elevated white blood cells, diagnosis of pneumonia, or urinary tract infection; (3) renal disease with glomerular filtration rate (GFR) < 45; and (4) recurrent stroke in the chronic stage, because these conditions could yield different metabolic profiles^13^.

### Metabolome analysis

A serum nuclear magnetic resonance (NMR) metabolomics platform (Nightingale Health Ltd, Helsinki, Finland) was used to quantify 271 circulating metabolites of non-lipidomic and lipidomic origin. This high-throughput metabolomics platform provides simultaneous quantification of amino acids, ketone bodies, glycolysis-related metabolites, routine lipids, abundant fatty acids, and lipid content of 14 lipoprotein subclasses, including small, medium, large, and very large high density lipoproteins (HDLs); intermediate density lipoproteins (IDLs); small, medium, and large low density lipoproteins (LDLs); and very small, small, medium, large, very large, and extremely large very low density lipoproteins (VLDLs)/chylomicrons in absolute concentration units. The measured variables include quantifications of absolute concentrations and selected ratios, primarily related to fatty acids and lipoprotein composition. The NMR platform has been applied extensively in epidemiological studies^14,15^, and details of the experiments have been described elsewhere^16,17^. Here we used NMR analysis in a cohort of stroke patients in acute and chronic stages, as well as controls.

### Statistical analysis

The clinical and demographic variables were summarized using mean with standard error for continuous variables and numbers and percentages for categorical variables. Before statistical analysis, the raw metabolite profile data were normalized through log-transformation, which resulted in some negative values. Analysis was performed in MetaboAnalyst 5.0. We used Sparse Partial Least Squares – Discriminant analysis (sPLS-DA) to inspect group disparity using data from all metabolites. We used a multivariate linear regression model to compare metabolites in (1) the acute stage of stroke vs. controls and (2) the chronic stage of stroke vs. controls; we used a mixed regression model to compare metabolites in (3) acute vs. chronic stages of stroke. We used age, sex, ethnicity, body mass index (BMI), smoking status, history of hyperlipidemia, and statin use as covariates to account for differences between stroke and control groups. The standard error for regression coefficient is reported. All regression p-values were corrected with False Discovery Rate (FDR).

## Results

**Figure 1.**
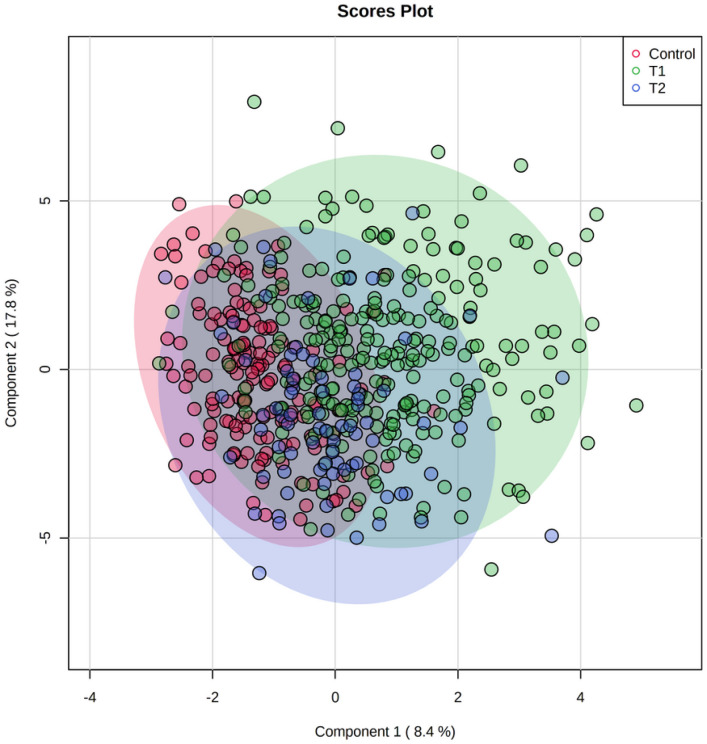
Sparse Partial Least Squares – Discriminant Analysis (sPLS-DA) for all evaluated metabolites. Three populations of metabolites: T1 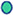 (green) – acute stage of stroke, T2 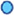 (blue)– chronic stage of stroke, controls without stroke 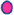 (red). The acute stage of stroke metabolites is visibly separated from the chronic stage and controls.

We analyzed data gathered on a cohort of 297 ischemic stroke patients and 159 controls without stroke. We collected serum samples on 297 stroke patients in acute and followed 137 patients to the chronic stage of stroke. Most patients in the stroke and control groups were Caucasian (67% and 82%), had a similar BMI of 30, and had a history of hypertension, hyperlipidemia, and coronary artery disease. Stroke patients were older (63 vs. 54 years of age) and more commonly smoked (46% vs. 23%). The average acute stage NIHSS was 8 ± 6.69, and average infarction volume was 28 ± 15 ml. (Table 1) In the acute stage, samples were collected on average 1.94 ± 0.99 days, and in the chronic stage 131.30 ± 73.40 days from last known well. sPLS-DA of all analyzed metabolites showed three distinct populations for the acute stage of stroke, the chronic stage of stroke, and controls. The differences were particularly evident for acute stage patients (Fig. 1).

Regression analysis with FDR correction in the three groups revealed that 38 compounds were elevated or decreased in stroke patients compared with controls, or in the acute stage of stroke compared with the chronic stage of stroke. Twenty of those compounds were of non-lipidomic, while 18 were of lipidomic origin. Five lipidomic compounds, including cholesterol, cholesteryl esters, phospholipids, triglycerides and total lipids, characterized the composition of high, intermediate, low, and very low-density lipoproteins, and are presented separately. Overall, we identified three trends in metabolome change: (1) metabolites elevated in the acute stage of stroke compared to controls, the chronic stage, or both; (2) metabolites elevated in the chronic stage of stroke compared with controls, the acute stage, or both; and(3) metabolites altered in stroke patients compared with controls, but without change from the acute stage to the chronic stage. Complete metabolome analysis of significant metabolites with levels, standard error, and p-values is presented in the supplementary table. For a better visual impression, Tables 2–4 only present the direction of change in significant metabolites (p < 0.05).

Analysis of significant non-lipidomic metabolites is presented in Table 2. Among ketone bodies, acetoacetate and beta-hydroxybutyrate were elevated in the acute stage compared with controls (0.44 ± 0.09; 0.6 ± 0.11) and the chronic stage (−0.13 ± 0.03; −0.11 ± 0.03); acetone was elevated in the acute (2.59 ± 0.32) and chronic stages (1.29 ± 0.44) compared with controls, and in the acute stage compared with the chronic stage (−0.33 ± 0.06); and acetate was elevated in the acute (1.11 ± 0.21) and chronic (0.46 ± 0.24) stages compared with controls. Among glycolysis metabolites, glycerol was elevated in the acute stage compared with controls (1.05 ± 0.27) and the chronic stage (−0.22 ± 0.07); pyruvate was elevated in the acute stage compared with controls (0.95 ± 0.21); lactate was decreased in stroke patients compared with controls (−0.84 ± 0.23; −1.09 ± 0.39), without change between the acute and chronic stages; and citrate was decreased in the chronic stage compared with controls (−2.13 ± 0.77). Among amino acids, alanine was decreased in the acute (−2.08 ± 0.4) and chronic stages (−1.82 ± 0.68) compared with controls, but increased from the acute to chronic stages of stroke (0.31 ± 0.14); histidine (−4.56 ± 0.62; −4.67 ± 0.94), glutamine (−2 ± 0.45; −1.37 ± 0.66), and tyrosine (−0.87 ± 0.39; −1.96 ± 0.69) remained low, while glycine(1.14 ± 0.31; 1.35 ± 0.53) and phenylalanine (3.38 ± 0.45; 1.5 ± 0.68) remained high in stroke patients compared with controls, without change between the acute and chronic stages. Branched chain amino acid (BCAA) isoleucine was elevated in the acute stage compared with controls (0.63 ± 0.26) and the chronic stage (−0.26 ± 0.1); leucin was elevated in the acute stage compared with the chronic stage (−0.27 ± 0.12); and valine was decreased in the acute (−0.83 ± 0.37) and chronic (−1.82 ± 0.65) stages of stroke compared with controls. Inflammatory marker glycoprotein acetyl was elevated in the acute stage compared with controls (1.09 ± 0.43).

Analysis of significant lipidomic metabolites is presented in Table 3. Phosphatidylcholines (−2.59 ± 0.45; −3.61 ± 0.77), total cholines (−2.5 ± 0.49; −3.98 ± 0.85), phosphoglycerides (−2.59 ± 0.45; −3.61 ± 0.77), and sphingomyelins (−1.16 ± 0.49; −3.97 ± 0.9) remained low in stroke patients compared with controls, without change from the acute to chronic stages. Total fatty acids (−1.38 ± 0.39; −3.29 ± 0.73), saturated fatty acids (−1.22 ± 0.36; −2.84 ± 0.66), monounsaturated fatty acids (−0.93 ± 0.32; −2.31 ± 0.57), and omega-6 fatty acids (−1.93 ± 0.47; −4.43 ± 0.91) were low in stroke patients compared with controls, and further decreased towards the chronic stage (total fatty acids − 0.33 ± 0.16; saturated fatty acids − 0.33 ± 0.15; monounsaturated fatty acids − 0.3 ± 0.13; omega-6 fatty acids − 0.36 ± 0.17). Polyunsaturated fatty acids (−1.73 ± 0.45; −3.93 ± 0.85) and linoleic acid (−1.77 ± 0.38; −3.71 ± 0.74) were lower in stroke patients than in controls, without change between the acute and chronic stages. Apolipoprotein A1 (−3.42 ± 0.53; −3.33 ± 0.85) and apolipoprotein B (−0.71 ± 0.34; −3.29 ± 0.67) were lower in stroke patients than in controls; apolipoprotein B further decreased in the chronic stage (−0.48 ± 0.12), although apolipoprotein A1 did not change.

Analysis of different lipoprotein subclasses is illustrated in Table 4. Overall, the content of cholesterol, cholesteryl esters, phospholipids, and triglycerides in most lipoprotein subclasses was either decreased or unchanged in acute stroke compared with controls, but significantly decreased towards the chronic stage.

## Discussion

In this study, we identified metabolites that are associated with AIS by simultaneous comparison of the serum metabolome in the acute stage of stroke with that of controls and the chronic stage of stroke. Overall, ketone bodies, BCAAs, energy, and inflammatory compounds were elevated/decreased in the acute stage and could have been driven by stroke acuity. In contrast, most amino acids, phosphatidylcholines, phosphoglycerides, and sphingomyelins were elevated/decreased in stroke patients compared with controls, but did not change between the acute and chronic stages, likely being unrelated to stroke acuity. These findings clarify changes in the metabolome between stages of stroke and may indicate a direction for future research investigating stroke biomarkers.

**Figure 2:**
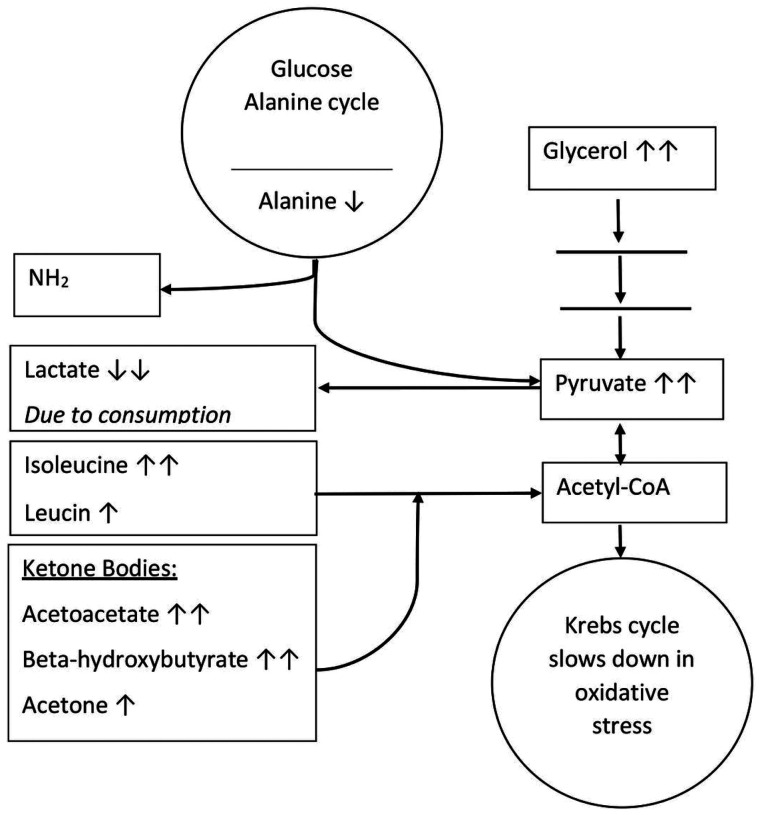
Metabolic pathway in ischemic stroke.

Our findings validated previously described changes in serum levels of ketone bodies, BCAAs, and energy compounds in acute stroke^4,18,19^. Involvement of these metabolite subclasses should come from interplay of different metabolic pathways, all triggered by cerebral vessel occlusion (Fig. 2). In our view, oxidative stress in the brain slows down the Krebs cycle; activates anaerobic glycolysis, leading to elevation of pyruvate and glycerol; and, at the same time, activates production of ketone bodies to support brain metabolic requirements. Acetone, acetoacetate, and β-hydroxybutyrate are more energetically efficient than glucose, and can provide up to 70% of the brain’s energy needs^20^. Further, β-hydroxybutyrate may ameliorate the disruption of cerebral energy metabolism after ischemia, when the anaerobic glycolytic pathway is activated^21^. In addition, studies suggest that cerebral uptake of ketones significantly increases during acute brain stress^20^. All these findings correspond to our observed increase in acetone, acetoacetate, β-hydroxybutyrate, pyruvate, and glycerol in the acute stage of stroke, followed by a decrease in the chronic stage to levels similar to controls. Acute ketosis may also increase levels of ketogenic amino acids by preventing their degradation to Acetyl-CoA, which, in our study, corresponds to an elevated acute level of isoleucine and relative decrease of leucin in the chronic stage^22^. Besides slowing of the Krebs cycle, pyruvate could have increased by synthesis from alanine through the glucose/alanine cycle, which also corresponds to decreased alanine levels. Although activation of anaerobic glucose metabolism is overall associated with increased lactate levels, previous experimental studies showed a decrease in lactate levels after stroke in the brain parenchyma^23^ and blood^24^, same as in our study. This decrease may be explained by utilization of lactate as an alternative energy source by still-viable brain tissue within the infarction^25^. Production of lactate from pyruvate may also be inhibited by β-hydroxybutyrate through inhibition by increased acetyl-CoA^21^. In the present study, however, decreased levels of lactate were observed in the acute and chronic stages of stroke, suggesting prolonged action of those mechanisms, or other, yet unknown, pathway.

Glycoprotein acetyl is an inflammatory biomarker previously associated with the risk of cardiovascular disease^26^. In the present study, glycoprotein acetyl was elevated in the acute stage of stroke compared with controls, but not in the chronic stage. Therefore, it may simply reflect acute reaction to stroke, rather than stroke risk.

Although in case-control studies AIS was associated with changes in metabolism of lipids and amino acids, the question of how long these changes continue could not be answered due to lack of follow-up data^2,27^. In the present study, follow-up (chronic stage) metabolome changes of lipids and most amino acids were similar to the acute stage. Although prolonged alteration of metabolism after stroke is possible, the absence of significant differences in lipids and most amino acid levels between the acute stage and the chronic stage is more logically explained by the baseline differences in metabolism, unrelated to stroke acuity. This corresponds to unconvincing pathophysiological explanations of amino acid and lipid metabolism after acute cerebral ischemia given in previous investigations^2^.

Serum levels of apolipoproteins, lipoproteins, and fatty acids are closely related to patient diet; therefore, their decrease may be explained by the dietary trends of stroke patients ^28^. Two major factors that affect diet after stroke are dysphagia and implementation of healthy lifestyles. Persistent dysphagia after stroke may lead to deficiencies of essential metabolites, such as omega-6 fatty acids, while healthier diets with decreased consumption of meat result in decreased saturated fatty acids and apolipoprotein B, and increased monounsaturated fatty acids. The latter was not observed in our study, likely because of patient failure to increase consumption of vegetables or dysphagia. In addition, some controls in our study were recruited from the cardiology clinic and could have diets rich in saturated fat, explaining higher levels of cholesterol in controls than in stroke patients.

Strengths of our study include its unique design in which the acute stage of stroke metabolome was compared with that of controls and the chronic stage, and the comparatively reasonable sample size. Validation of data from other laboratories that used liquid chromatography mass-spectroscopy for metabolome analysis adds to the credibility of our results. Limitations include a comparatively low follow-up rate, some inequalities between the stroke and control groups, and the lack of long-term follow-up data. Using multiple covariates for regression analysis helped to address disparities in the stroke and control groups, but at the same time could significantly change the results of our or any other metabolome study. Particularly, adjusting for use of statins affected lipidomic compounds. In the future, development of standardized protocols for evaluation of the metabolome may help to improve interpretation of the results.

In summary, our pilot study showed differences in metabolism between stroke patients in the acute and chronic stages and controls, and helped to differentiate metabolites altered in the acute stage of stroke from those altered in the acute and chronic stages. Further validation of these findings in a larger, independent cohort is needed to provide a solid background for future stroke metabolome research.

## Figures and Tables

**Table 1:** Baseline demographic and clinical characteristics of ischemic stroke cohort and controls

Characteristic	Stroke Patients (n = 297)	Controls (n = 159)	p-value
	Mean ± Standard error for continuous, and number (%) for categorical variables	Mean ± Standard error for continuous variables; number (%) for categorical variables	
Age	63.21 ± 0.82	54.84 ± 1.30	< 0.01
Weight (Kg)	88.31 ± 1.28	90.92 ± 2.49	0.33
Height (cm)	170.49 ± 0.59	169.53 ± 0.98	0.43
Glucose	141.81 ± 4.41	106.47 ± 3.64	< 0.01
A1c	6.71 ± 0.21	5.0 ± 0.0	0.05
HDL	44.14 ± 0.75	49.08 ± 1.65	0.01
LDL	104.54 ± 2.56	94.50 ± 5.27	0.13
Cholesterol	177.0 ± 3.02	166.92 ± 6.26	0.19
Triglycerides	146.52 ± 6.07	119.69 ± 8.13	0.09
BMI	30.29 ± 0.40	30.27 ± 0.57	0.97
Female	158 (51 %)	98 (61 %)	0.02
Caucasian	210 (67%)	130 (82%)	< 0.01
African American	69 (22%)	11 (7%)	
Asian or Native American	34 (11 %)	18 (11 %)	
History of hyperlipidemia	121 (38%)	49 (31 %)	0.09
History of coronary artery disease	67 (21 %)	33 (21 %)	0.87
History of smoking	143 (46%)	37 (23%)	< 0.01
History of hypertension	220 (70%)	97 (61 %)	0.10
